# Behavioral and neurocognitive factors distinguishing post-traumatic stress comorbidity in substance use disorders

**DOI:** 10.1038/s41398-023-02591-3

**Published:** 2023-09-14

**Authors:** David C. Houghton, Heidi M. Spratt, Lori Keyser-Marcus, James M. Bjork, Gretchen N. Neigh, Kathryn A. Cunningham, Tatiana Ramey, F. Gerard Moeller

**Affiliations:** 1 Center for Addiction Sciences and Therapeutics, University of Texas Medical Branch, Galveston, TX USA; 2 Department of Psychiatry and Behavioral Sciences, University of Texas Medical Branch, Galveston, TX USA; 3 Department of Biostatistics and Data Science, University of Texas Medical Branch, Galveston, TX USA; 4 Institute for Drug and Alcohol Studies, Virginia Commonwealth University, Richmond, VA USA; 5 Department of Anatomy and Neurobiology, Virginia Commonwealth University, Richmond, VA USA; 6 Department of Pharmacology and Toxicology, University of Texas Medical Branch, Galveston, TX USA; 7 Division of Therapeutics and Medical Consequences, National Institute of Drug Abuse, National Institutes of Health, Rockville, MD USA

**Keywords:** Diagnostic markers, Neuroscience

## Abstract

Significant trauma histories and post-traumatic stress disorder (PTSD) are common in persons with substance use disorders (SUD) and often associate with increased SUD severity and poorer response to SUD treatment. As such, this sub-population has been associated with unique risk factors and treatment needs. Understanding the distinct etiological profile of persons with co-occurring SUD and PTSD is therefore crucial for advancing our knowledge of underlying mechanisms and the development of precision treatments. To this end, we employed supervised machine learning algorithms to interrogate the responses of 160 participants with SUD on the multidimensional NIDA Phenotyping Assessment Battery. Significant PTSD symptomatology was correctly predicted in 75% of participants (sensitivity: 80%; specificity: 72.22%) using a classification-based model based on anxiety and depressive symptoms, perseverative thinking styles, and interoceptive awareness. A regression-based machine learning model also utilized similar predictors, but failed to accurately predict severity of PTSD symptoms. These data indicate that even in a population already characterized by elevated negative affect (individuals with SUD), especially severe negative affect was predictive of PTSD symptomatology. In a follow-up analysis of a subset of 102 participants who also completed neurocognitive tasks, comorbidity status was correctly predicted in 86.67% of participants (sensitivity: 91.67%; specificity: 66.67%) based on depressive symptoms and fear-related attentional bias. However, a regression-based analysis did not identify fear-related attentional bias as a splitting factor, but instead split and categorized the sample based on indices of aggression, metacognition, distress tolerance, and interoceptive awareness. These data indicate that within a population of individuals with SUD, aberrations in tolerating and regulating aversive internal experiences may also characterize those with significant trauma histories, akin to findings in persons with anxiety without SUD. The results also highlight the need for further research on PTSD-SUD comorbidity that includes additional comparison groups (i.e., persons with only PTSD), captures additional comorbid diagnoses that may influence the PTSD-SUD relationship, examines additional types of SUDs (e.g., alcohol use disorder), and differentiates between subtypes of PTSD.

## Introduction

The association between substance use disorders (SUDs) and post-traumatic stress is well-established [1–4]. Approximately 30–60% of persons seeking treatment for SUDs also meet diagnostic criteria for post-traumatic stress disorder (PTSD) [5, 6], and those with comorbid presentations typically demonstrate a more severe clinical profile [7] with poorer treatment outcomes [8, 9]. Several functional explanations for this association have been put forth [10]. For instance, substance use may serve as a “self-medication” strategy for acutely attenuating post-traumatic stress [11], while ironically worsening emotional health and exacerbating PTSD symptoms [12]. To interrupt this negative cycle, integrative treatments have been developed that focus on mitigating maladaptive avoidance behaviors and improving coping skills [13–15]. Unfortunately, while these integrative treatments effectively reduce PTSD symptoms, they show limited efficacy to reduce substance use [16–20].

A clear understanding of the hallmark risk factors and distinct endophenotypic profiles is needed to advance treatment strategies for trauma-exposed SUD populations. Various neurobehavioral risk factors have been linked with comorbid SUD and PTSD, including increased anxiety sensitivity [21], anhedonia [22, 23], impulsivity [24–28], more severe executive functioning deficits [29–31], and increased sensitivity to reward and punishment [32]. Yet, while such evidence supports potential etiological pathways toward shared PTSD-SUD vulnerability, the neurobehavioral traits that consistently distinguish comorbid from non-comorbid patients have not yet been identified. Machine learning (ML) is an analytic approach well suited to this task [33–37] and has been utilized by our research group to delineate a multivariate neurobehavioral profile of cocaine dependence [38]. Indeed, ML techniques have considerable advantages over traditional inferential case-control comparisons, including superior accuracy, the ability to handle numerous (and intercorrelating) predictors simultaneously, and the capacity to quantify the relative importance of individual predictors [39].

In the present study, we employed supervised ML to determine neurobehavioral phenotypic features that associated with post-traumatic stress symptoms within a SUD population. Candidate predictors were gathered using the standardized Phenotyping Assessment Battery (PhAB) constructed in collaboration with the National Institute of Drug Abuse (NIDA) [40] based on Research Domain Criteria principles [41, 42] for deployment in clinical trials of SUD pharmacotherapies [43]. The PhAB is a modular compendium of neurofunctional domain-based assessments [40]. Our study sought to identify comorbidity predictors from each of: the negative emotionality domain (e.g., low distress tolerance, anhedonia, anxiety, depression), metacognitive domain (e.g., distorted thought patterns and beliefs), reward domain (e.g., impulsivity, sensitivity to threat), interoceptive domain (e.g., willingness to tolerate aversive body sensations), and sleep domain (e.g., poor sleep quality). In addition, a subset of participants were administered a battery of neurocognitive measures to assess the predictive value of cognitive functioning, including working memory, attention, delay discounting, motoric impulsivity (i.e., response inhibition), and attentional bias towards threat. Consistent with our goal of identifying a neurobehavioral profile of comorbid post-traumatic stress symptoms in SUD, we constructed a series of classification and regression trees predicting post-traumatic stress symptom severity (i.e., score on the Post-traumatic Stress Disorder Checklist for DSM-5 (PCL-5)) as well as binary group identification (i.e., significant comorbid PTSD symptoms vs. no significant comorbid PTSD symptoms).

## Materials and methods

The Virginia Commonwealth University Institutional Review Board approved the study and written informed consent was obtained from all participants. Data for the present investigation were collected in the course of a feasibility study for the NIDA PhAB [40].

### Subjects

Participants were recruited for the parent study from an established participant registry and through local advertising [40]. Eligibility criteria were intentionally broad to recruit a “real-world” sample of individuals with SUD. To be eligible for participation, individuals were required to be: (a) between 18 and 70 years of age, (b) able to complete forms and interviews in English, and (c) meet DSM-5 diagnostic criteria for a current, primary SUD with the substance of choice being either cocaine, marijuana, or opioids. The MINI International Neuropsychiatric Interview Version 7.0.2 [44] was used to determine DSM-5 SUD diagnoses. The presence of any other condition or illness that, in the opinion of the Principal Investigator or study physician, would preclude safe and/or successful completion of the study was also a cause for exclusion. Participants could not meet DSM-5 criteria for a severe SUD related to other substances. However, due to the ubiquity of polysubstance use in the local population, individuals with mild to moderate comorbid SUD (i.e., alcohol use disorder) were not excluded. Confirmatory urine drug toxicology screens and alcohol breath samples were used in conjunction with self-report to assess recent substance use. Participants were excluded if they met any of the following criteria: (a) current psychosis, mania, suicidal or homicidal ideation, (b) history of seizures (excluding childhood febrile seizures), or (c) loss of consciousness from traumatic brain injury for more than 30 min. A total of 160 participants met inclusion/exclusion criteria and completed all self-report measures, while 102 participants completed additional neurocognitive measures. Participant characteristics are shown in Table 1, and there were no differences between groups with regard to age, sex, or drug of choice.

**Table 1:** Demographic characteristics of participants.

	Total	No/low trauma	Moderate/severe trauma	Test statistic (*t*/*X*^2^)	*p* value
*Total sample*					
Age (years)					
Mean (SD)	42 (13)	43 (14)	41 (13)	0.56	0.58
Sex					
Female	69 (43%)	50 (45%)	19 (39%)	0.55	0.46
Male	91 (57%)	61 (55%)	30 (61%)		
Participant group					
Cocaine use disorder	37 (23%)	28 (25%)	9 (18%)	1.19	0.55
Marijuana use disorder	47 (29%)	31 (28%)	16 (33%)		
Opioid use disorder	76 (48%)	52 (47%)	24 (49%)		
Race					
Black or African American	121 (76%)	88 (79%)	33 (67%)	5.92	0.31
White	34 (21%)	20 (18%)	14 (29%)		
Other/declined to answer	5 (3%)	3 (3%)	2 (4%)		
*n*	160	111	49		
*Neurocognitive sub-sample*					
Age (years)					
Mean (SD)	44 (11)	45 (11)	44 (11)	0.48	0.63
Sex					
Female	41 (40%)	31 (44%)	10 (31%)	1.55	0.21
Male	61 (60%)	39 (56%)	22 (69%)		
Participant group					
Cocaine use disorder	22 (22%)	15 (21%)	7 (22%)	0.89	0.64
Marijuana use disorder	25 (25%)	19 (27%)	6 (19%)		
Opioid use disorder	55 (54%)	36 (51%)	19 (59%)		
Race					
Black or African American	81 (79%)	56 (80%)	25 (78%)	0.59	0.75
White	20 (20%)	13 (19%)	7 (22%)		
Other/declined to answer	1 (1%)	1 (1%)	0 (0%)		
*n*	102	70	32		

### Assessments

#### Assessment-derived predictor variables

The NIDA PhAB “core” SUD-relevant domains evaluate cognition, reward, interoception, negative emotionality, metacognition, and sleep [40] (See Supplemental Information for fuller details on questionnaires and cognitive tasks). Nine self-report questionnaires and five neurocognitive tasks were employed here (Table 2). Each measure within the PhAB produced between one and eight scale/subscale scores. The battery of questionnaires/tasks was administered electronically via Redcap and Inquisit Lab software (Millisecond Software LLC, Seattle WA), and the average completion time for the battery was 180 min.

**Table 2:** PhAB assessments.

Assessment	Predictor variable	Description of variable
Questionnaire-based assessments		
Short UPPS	Negative Urgency (NURG)	Tendency to act impulsively under conditions of negative affect
	(Lack of) Premeditation (PREM)	Tendency to act without planning or without consideration of potential consequences
	(Lack of) Perseverance (PERS)	Tendency to give up in the face of adverse circumstances
	Sensation Seeking (SS)	Interest in an tendency to pursue novel and exciting activities
	Positive Urgency (PURG)	Tendency to give in to impulses under conditions of high positive affect
Multidimensional Assessment of Interoceptive Awareness	Noticing	Awareness of body sensations
	Not Distracting	(Lack of) tendency to ignore or distract oneself from aversive body sensations
	Not worrying	(Lack of) distress or worry in response to aversive body sensations
	Attention Regulation	Ability to focus on body sensations
	Emotional Awareness	Recognition of the connection between body sensations and emotional states
	Self-Regulation	Ability to modulate emotion in response to bodily sensations
	Body Listening	Tendency to focus on body sensations to determine how one is feeling
	Trusting	Overall feeling of comfort and safety in one’s body
Distress Tolerance Scale	General (G)	Overall distress tolerance (higher scores reflect poorer distress tolerance)
	Tolerance	Tendency to perceive distress as unbearable
	Absorption	Tendency to become overwhelmed by strong emotions
	Appraisal	Perceived ability to accept strong emotions
	Regulation	Ability to manage distressing feelings
PROMIS—Depression	Total *t*-score	Depression severity
PROMIS—Anxiety	Total *t*-score	Anxiety severity
Buss–Perry Aggression Scale	Total Score	Overall index of aggression
	Physical Aggression	Tendency to engage in physical aggression
	Verbal Aggression	Tendency to engage in verbal aggression
	Anger	Severity of aggressive feelings
	Hostility	Propensity for paranoia and suspicion toward others
Snaith–Hamilton Pleasure Scale	Total Score	Level of anhedonia (high scores indicate higher anhedonia)
Metacognitions Questionnaire	Total Score	Overall level of perseverative thinking, biased attention, and ineffective self-regulation strategies
	Cognitive Confidence	Trust in one’s memory
	Positive Beliefs	Belief that worry is helpful
	Cognitive Self-Consciousness	Degree of self-monitoring over thoughts
	Uncontrollability/Danger	Ability to control and regulate worrisome thoughts
	Need to Control Thoughts	Belief that thoughts must be controlled
Pittsburg Sleep Quality Index	Total Score	Overall Sleep Efficiency
Neurocognitive assessments		
Backwards Digit Span	Two-error maximum length (bTE_ML)	Index of working memory ability
	Two-error total trials (bTE_TT)	Consistency in working memory testing
	Maximal backward digit span (bML)	Cumulative number of correct recalls during testing
	Mean digit span (MS)	Average number of digits recalled
Emotional Go-NoGo Task	Commission error rate	Motoric impulsivity: proclivity for failure to withhold responding to non-target facial expression type.
	Fear Effect RT calculated as: medianRT_CF_ − median RT_FC_	Motivational salience of fear stimuli: Degree to which fear faces as targets speed reaction time (RT)
	Happy Effect RT calculated as: medianRT_CH_ − medianRT_HC_	Motivational salience of happy stimuli: Degree to which happy faces as targets speed reaction time (RT)
	Fear Effect FA calculated as: FArate_CF_ − FArate_FC_	Cognitive disruption by fear stimuli: Degree to which fear faces as non-targets increase commission errors
	Happy Effect FA calculated as: FArate_CH_ − FArate_HC_	Cognitive disruption by happy stimuli: degree to which happy faces as non-targets increase commission errors
Attentional Network Task	Alerting Effect	Ability to leverage timing information about target
	Orienting Effect	Ability to leverage spatial information about target
	Conflict Effect	Vulnerability to incongruent flanking stimuli to slow RT
5-Trial Delay Discounting	Effective Delay 50% (ED50)	The estimated delay at which the value of the offered reward was discounted by 50% by having to wait
	Discounting constant *k*	Severity of discounting of subjective reward value with delay to delivery (high scores denote greater impulsivity)
Stop Signal Task	Stop signal delay (SSD)	Mean interval in ms between “go” and subsequent “stop” signal that fostered a 50% successful rate of stopping
	Stop signal reaction time (SSRT)	Time required to stop an initiated go process (smaller numbers reflect better inhibition)
	Mean reaction time in stop trials (SR_RT)	Response times of commission errors in stop trials
	Mean reaction time in go trials (NS_RT)	Response times during correct “go” responses
	Hit percentage in go trials (NS_HIT)	Percent correct indication of direction (L/R) of target arrow
	Miss percentage (NS_MISS)	Percentage of “go” trials to which there was no response

#### Assessment-derived outcome definitions

Because trauma exposure itself or even subthreshold PTSD symptoms may complicate SUD presentation [45] or treatment [46], the source PhAB protocol administered a dimensional probe of PTSD symptomatology using the PCL-5 [47]. The PCL-5 is a 20-item self-report questionnaire that assesses key DSM-5 criteria for PTSD. Items are rated on a 5-point Likert scale ranging from 0 to 4, and item ratings are summed to produce total scores ranging from 0 to 80 with higher scores reflecting greater symptom severity. Because most previous reports on PTSD-SUD comorbidity utilized clinical diagnostic criteria to obtain a binary (present/absent) diagnosis of PTSD, for harmonization with these studies, our analyses (described below) also featured a proxy dichotomization of (subclinical) vs. clinically-significant PTSD in addition to a continuous PTSD dimensional score. Based on previous findings [48], persons with scores ≥33 tend to have moderate to severe PTSD symptoms and probably meet DSM-5 criteria for PTSD, whereas persons with scores <33 tend to exhibit either a low level of PTSD symptoms or no PTSD symptoms. Thus, to demarcate participants with clinically significant comorbid PTSD, we employed this empirically derived cutoff score of 33. Below a score of 33, the participant was classified with “absent to mild” PTSD symptoms. At or above 33, the participant was classified with “clinically significant” PTSD symptoms. Thus, this binary outcome was employed for supervised ML. The effect sizes of association between each predictor variable and the target variable (no significant PTSD symptoms vs. significant PTSD symptoms) are shown in Supplementary Figs. 1 and 2.

### Statistical analysis

We used *R* version 4.0.3 and Salford Predictive Modeler (SPM) version 8 to perform all statistical analyses, and 0.05 was set as the level of significance for all relevant hypothesis tests. The scores from the PhAB battery described above comprised an initial 72 explanatory (predictor) variables. Due to technical errors and/or participant failures to comply with task instructions, complete and valid neurocognitive task data were available from 102 participants. Our primary analysis centered on the full sample (*n* = 160) for whom complete questionnaire data were available. Subsequently, we performed follow-up analyses using data from the 102-participant subset (Table 1) wherein the neurocognitive performance variables (Table 2) were added to the model with the questionnaire-based metrics (Table 2). We were, therefore, able to infer whether neurocognitive metrics emerged as appreciable, i.e., top 20 predictors of PTSD symptomotology and whether their inclusion improved the prediction of PTSD symptom status.

The ML algorithms employed in the study were TreeNet and Classification and Regression Trees (CART). TreeNet [49] is an empirical variable selection procedure that can be used to efficiently reduce the number of explanatory variables in a predictive model. TreeNet algorithms [50, 51], also known as stochastic gradient boosting, offer several unique and useful features. These include (a) built-in estimation of prediction accuracy, (b) measures of feature importance, and (c) a measure of similarity between sample inputs. TreeNet improves upon classical decision trees such as CART [52] with retention of the most appealing properties of tree methods. The product of TreeNet is a ranked list of variable importance which is based on predictive models from an ensemble of weak learners in the form of classification trees. This methodology frequently outperforms Random Forest methods in prediction and variable selection [53]. “Boosting” is a method that seeks to convert weak learners into stronger ones. Here, a learner is an algorithm, and in the case of stochastic gradient boosting, that algorithm is CART; thus, boosting aims to improve on CART methods by creating multiple CART trees based on subsets of the data. The result is that TreeNet creates a final model in a gradual, additive, and sequential manner. All default options for SPM were used, and the algorithm was run with 80% of the data randomly selected for model training, and 20% of the data randomly selected for model testing. Based on the TreeNet results, the top 20 most important variables were used as predictor variables for the CART algorithm [54], which was used to predict the target outcome.

CART is a highly useful nonparametric method for building decision trees and predicting novel relationships between phenotypic predictors and biomedical outcomes because there is no requirement to select input variables based on theoretical importance. CART handles structurally complex datasets comprised of both categorical and continuous data with extreme robustness and limited vulnerability to outliers [49]. The three main components of CART involve creating a set of rules for splitting each node in a tree, deciding when a tree is fully grown, and assigning an outcome prediction to each terminal node of the tree [55]. Decision trees produce a clearly interpretable split at each node which is a binary response of some feature in the data set. The basic algorithm for building the decision tree seeks a data feature which maximizes the split between the classes contained in the parent node. CART is a recursive algorithm such that, once an appropriate split resulting in two child nodes is determined, the child nodes then become the new parent nodes, and the process is carried on down the branches of the tree. The CART tree is considered fully grown once a split cannot be identified that reduces model impurity. CART uses cross-validation techniques to determine the accuracy of the decision trees.

We first derived CART models based on a regression approach in which the target variable was the continuous score on the PCL-5. In a regression-based decision tree, the continuous target variable is split using recursive partitioning into a series of bins containing subsets of scores along the continuum. The algorithm does not attempt to predict the exact score on the target variable for each individual, but rather the bin into which that individual’s target score belongs. The average target score within each bin is therefore used as the predicted target score for all individuals who are presumed to belong in the respective bins. The number of bins and boundaries between bins are determined based on quantitative cross-validation aimed at reducing error. For instance, a small number of large bins typically diminishes precision, whereas a large number of small bins increases precision, but can sometimes result in overfitting and insufficient generalizability. We evaluated the accuracy of regression-based models using goodness-of-fit, or *R*^2^.

We subsequently derived classification-based CART models, which attempted to classify participants with subclinical levels PTSD symptoms vs. those with clinically-significant levels of PTSD symptoms. As such, the outcome categories were set using the PCL-5 trauma score split into a binary variable based on the empirically derived cutoff at 33. Model performance was examined using confusion matrices, sensitivity and specificity, the F1-score (harmonic mean of precision and recall), as well as area under the receiver operating characteristic (ROC) curve. Based on established benchmarks for the predictive accuracy of psychological tests within the literature [56], we deemed 70% accuracy to reflect acceptable performance, 80% accuracy to reflect good performance, and 90% accuracy to reflect excellent performance.

## Results

### Full sample analysis

#### Regression

**Fig. 1:**
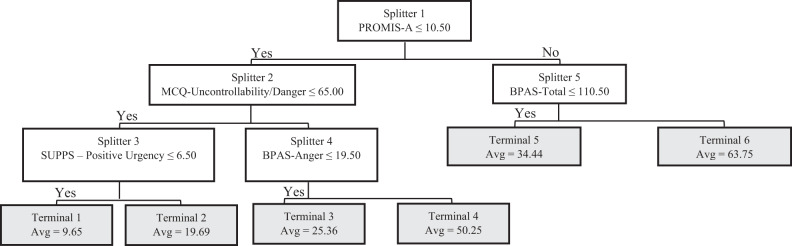
CART decision tree—regression—full sample. White rectangles represent intermediate nodes (i.e., cohorts that could be further split); gray rectangles represent terminal nodes; PROMIS-A PROMIS Anxiety, MCQ-UD Metacognitions Questionnaire—Uncontrollability/Danger, SUPPS Short Urgency-Premeditation-Perseverance-Sensation Seeking-Positive Urgency Impulsive Behavior Scale, BPAS Buss–Perry Aggression Scale.

The goodness-of-fit for the TreeNet training and testing datasets were 0.88 and 0.77, respectively, thus indicating an acceptable degree of explained variance. The 20 most important predictor variables identified by the TreeNet algorithm (Table 3), and the resulting CART algorithm produced a tree containing five primary parent (i.e., splitting) nodes and six child (i.e., terminal) nodes (Fig. 1). Variables used as splitting nodes, in order of importance, were the PROMIS Anxiety, Metacognitions Questionnaire—Uncontrollability/Danger, Buss–Perry Total Score, Buss–Perry Anger, and SUPPS Positive Urgency. Unfortunately, this model failed to demonstrate adequate goodness-of-fit, with *R*^2^ values in the training and testing datasets at 0.59 and 0.42, respectively.

**Table 3:** Top 20 predictors of significant PTSD symptoms in TreeNet Regression analysis—full sample.

Rank	Construct	Scale-subscale	Relative importance
1	Anxiety	PROMIS-A	100.00
2	Metacognitions	MCQ-Uncontrollability/Danger	71.59
3	Depression	PROMIS-D	55.42
4	Impulsivity	SUPPS-Positive Urgency	51.57
5	Metacognitions	MCQ-Total Score	45.38
6	Distress Tolerance	DTS-Tolerance	43.43
7	Interoceptive Awareness	MAIA-Not Distracting	39.22
8	Impulsivity	SUPPS-Negative Urgency	38.08
9	Distress Tolerance	DTS-Total Score	36.31
10	Aggression	BPAS-Total Score	35.55
11	Aggression	BPAS-Anger	29.56
12	Interoceptive Awareness	MAIA-Not Worrying	29.56
13	Aggression	BPAS-Physical Aggression	28.39
14	Anhedonia	SHAPS	28.28
15	Distress Tolerance	DTS-Absorption	27.92
16	Metacognitions	MCQ-Cognitive Self-Consciousness	27.61
17	Aggression	BPAS-Hostility	25.67
18	Interoceptive Awareness	MAIA-Emotional Awareness	22.11
19	Metacognitions	MCQ-Cognitive Confidence	20.91
20	Sleep Quality	PSQI	20.71

#### Classification

**Fig. 2:**
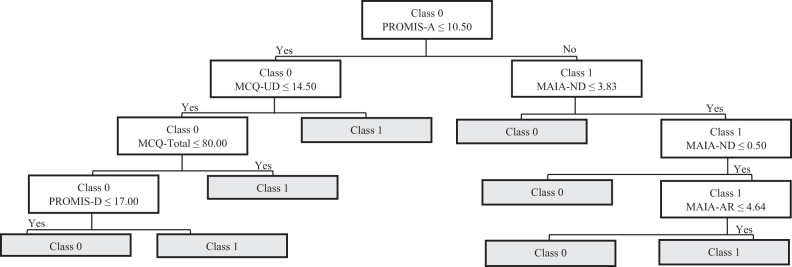
CART decision tree—classification—full sample. White rectangles represent intermediate nodes (i.e., cohorts that could be further split); gray rectangles represent final nodes; Class 0 = PCL-5 < 33; Class 1 = PCL-5 ≥ 33; PROMIS-A PROMIS Anxiety, MCQ-UD Metacognitions Questionnaire—Uncontrollability/Danger, MAIA-ND Multidimensional Assessment of Interoceptive Awareness—Not Distracting, PROMIS-D PROMIS Depression, MAIA-AR Multidimensional Assessment of Interoceptive Awareness—Attention Regulation.

Model accuracy for the TreeNet training and testing datasets was 93.88 and 73.88%, respectively, indicating that the variable selection process performed well. Indeed, the examination of the areas under the ROC curves showed good to excellent predictive accuracy, with the value for the training data at 0.97 (sensitivity = 97.44%, specificity = 90.32%) and the value for the testing data at 0.84 (sensitivity = 80.00%, specificity = 66.67%). Confusion matrices and ROC curves for TreeNet analysis are presented in Supplementary Material (Supplementary Tables 1 and 2 and Supplementary Fig. 3). The 20 most important predictor variables identified by the TreeNet algorithm are shown in Table 4. Accuracies for the CART training and testing algorithms were 92.80% (sensitivity = 97.44%, specificity = 87.10%; F1 = 86.36%) and 76.11% (sensitivity = 80.00%, specificity = 72.22%; F1 = 69.57%), respectively (confusion matrices shown in Supplementary Tables 3 and 4). Areas under the ROC curves for both training and testing data (Supplementary Fig. 3) were 0.94 and 0.78, respectively. These results indicate slight model overfitting for the training data. The decision tree derived in CART is displayed in Fig. 2. Variables used as splitting nodes in the CART algorithm consisted of the following (in order of importance): PROMIS Anxiety, Metacognition Questionnaire—Uncontrollability/Danger, Multidimensional Assessment of Interoceptive Awareness—Not Distracting, PROMIS Depression, Metacognition Questionnaire—Total Score, and Multidimensional Assessment of Interoceptive Awareness—Attention Regulation.

**Table 4:** Top 20 predictors of significant PTSD symptoms in TreeNet classification analysis—full sample.

Rank	Construct	Scale-subscale	Relative importance
1	Anxiety	PROMIS-A	100.00
2	Depression	PROMIS-D	68.57
3	Metacognition	MCQ-Uncontrollability/Danger	55.98
4	Metacognition	MCQ-Total Score	53.57
5	Metacognition	MCQ-Cognitive Self-Consciousness	42.44
6	Distress Tolerance	DTS-Tolerance	41.76
7	Interoceptive Awareness	MAIA-Not Distracting	39.27
8	Interoceptive Awareness	MAIA-Not Worrying	35.42
9	Interoceptive Awareness	MAIA-Trusting	32.53
10	Impulsivity	S-UPPS-Positive Urgency	32.11
11	Aggression	BPAS-Hostility	27.44
12	Distress Tolerance	DTS-Total Score	25.03
13	Anhedonia	SHAPS	24.01
14	Sleep Quality	PSQI	23.83
15	Interoceptive Awareness	MAIA-Attention Regulation	23.69
16	Metacognition	MCQ-Cognitive Confidence	23.07
17	Impulsivity	S-UPPS-Negative Urgency	22.55
18	Aggression	BPAS-Total Score	22.52
19	Impulsivity	S-UPPS-Perseverance	21.84
20	Aggression	BPAS-Anger	19.28

### Neurocognitive sub-sample analysis

#### Regression

**Fig. 3:**
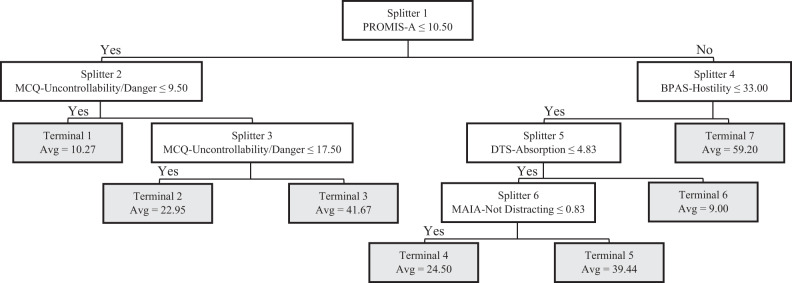
CART decision tree—regression—neurocognitive sample. White rectangles represent intermediate nodes (i.e., cohorts that could be further split); gray rectangles represent terminal nodes; PROMIS-A PROMIS Anxiety, MCQ Metacognition Questionnaire, BPAS Buss–Perry Aggression Scale, DTS Distress Tolerance Scale, MAIA Multidimensional Assessment of Interoceptive Awareness.

The goodness-of-fit for the TreeNet training and testing datasets were 0.69 and 0.64, respectively, thus indicating slightly sub-optimal model performance. The twenty most important predictor variables identified by the TreeNet algorithm are shown in Table 5. Results demonstrated that some neurocognitive task metrics supplanted self-report scales as predictors of trauma symptomatology, namely orienting and conflict effects in the Attention Network Task and happy and fear effects in the Emotional Go-NoGo Task (See Supplemental Material for detailed descriptions of neurocognitive task metrics). The subsequent CART algorithm produced a tree containing six primary splitting nodes and seven terminal nodes (Fig. 3). Variables used as splitting nodes, in order of importance, included PROMIS Anxiety, Metacognitions Questionnaire—Uncontrollability/Danger, Buss-Perry Hostility, Distress Tolerance Scale—Absorption, and Multidimensional Assessment of Interoceptive Awareness—Not Distracting. No neurocognitive assessments emerged as splitting nodes. This model demonstrated potentially adequate goodness-of-fit, with *R*^2^ values in the training and testing datasets at 0.72 and 0.64, respectively.

**Table 5:** Top 20 predictors of significant PTSD symptoms in TreeNet regression analysis—neurocognitive sub-sample.

Rank	Construct	Scale-subscale	Relative importance
1	Depression	PROMIS-D	100.00
2	Anxiety	PROMIS-A	93.39
3	Metacognition	MCQ-Uncontrollability/Danger	84.81
4	Distress Tolerance	DTS-Total Score	73.24
5	Distress Tolerance	DTS-Tolerance	38.47
6	Metacognition	MCQ-Cognitive Confidence	38.40
7	Metacognition	MCQ-Total	37.36
8	Anhedonia	SHAPS	37.24
9	Attentional Bias – Fear	EGNG-Fear Effect	35.70
10	Distress Tolerance	DTS-Absorption	34.05
11	Impulsivity	SUPPS-Positive Urgency	31.76
12	Aggression	BPAS-Hostility	30.76
13	Sleep Quality	PSQI	27.16
14	Interoceptive Awareness	MAIA-Not Distracting	27.08
15	Attentional Bias – Happiness	EGNG Happy Effect	25.98
16	Aggression	BPAS-Physical Aggression	25.93
17	Attention	ANT-Orienting Effect	25.92
18	Interoceptive Awareness	MAIA-Not Worrying	25.64
19	Impulsivity	SUPPS-Negative Urgency	20.66
20	Aggression	BPAS-Total Score	19.61

#### Classification

**Fig. 4:**
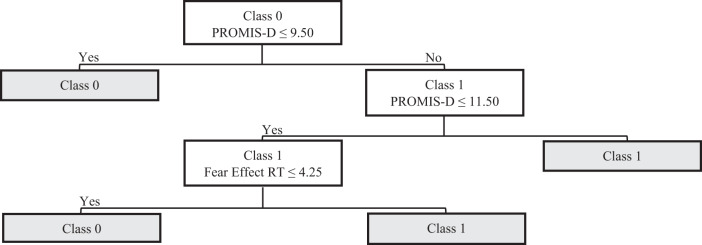
CART decision tree—neurocognitive sub-sample. White rectangles represent intermediate nodes (i.e., cohorts that could be further split); gray rectangles represent final nodes; Class 0 = PCL-5 < 33; Class 1 = PCL-5 ≥ 33; PROMIS-D PROMIS Depression, Fear Effect RT Fear effect reaction time from Emotional Go-NoGo Task.

Within the 102-participant sub-sample who completed all questionnaires and neurocognitive tasks, supervised ML analysis demonstrated good performance in predicting the presence vs. absence of clinically-significant ratings of PTSD symptoms. Model accuracy for TreeNet training and testing datasets were 86.21 and 73.33%, respectively (confusion matrices are shown in Supplementary Tables 5 and 6). Predictive performance, as evidenced by areas under the receiving operator characteristic curves, were 0.98 for the training data and 0.78 for the testing data (Supplementary Fig. 5). Although results indicated model overfitting for the training data, model accuracy was high enough to warrant further analysis. The 20 most important variables identified by the TreeNet algorithm, which included some neurocognitive assessments, are shown in Table 6. With the top 20 variables (self-report *plus* cognitive performance) entered as predictors, accuracies for the CART training and testing algorithms were 89.66% (Sensitivity = 100%; Specificity = 79.31%; F1 = 82.86%) and 86.67% (Sensitivity = 66.67%; Specificity = 75.00%; F1 = 41.39%) (confusion matrices shown in Supplementary Tables 7 and 8). Predictive accuracy, as reflected in an area under the curve, was 0.93 for the training data and 0.81 for the testing data (Supplementary Fig. 6). These results indicate good to excellent predictive accuracy. The decision tree derived in this CART analysis is displayed in Fig. 4. The only variables that emerged as splitting nodes in this CART algorithm consisted of the PROMIS Depression and the Fear Effect from the Emotional Go-NoGo Task (in order of importance).

**Table 6:** Top 20 predictors of significant PTSD symptoms in TreeNet classification analysis—neurocognitive sub-sample.

Rank	Construct	Scale-subscale	Relative importance
1	Depression	PROMIS-D	100.00
2	Anxiety	PROMIS-A	81.60
3	Aggression	BPAS-Hostility	40.71
4	Interoceptive Awareness	MAIA-Not Distracting	38.73
5	Metacognition	MCQ-Cognitive Confidence	33.84
6	Sleep Quality	PSQI	28.75
7	Impulsivity	S-UPPS-Perseverance	27.04
8	Response Inhibition	SST-NS_HIT	25.23
9	Metacognition	MCQ-Total Score	23.97
10	Metacognition	MCQ-Uncontrollability/Danger	21.56
11	Attention	ANT-Orienting Effect	21.39
12	Interoceptive Awareness	MAIA-Attention Regulation	18.71
13	Aggression	BPAS-Physical Aggression	17.87
14	Metacognition	MCQ-Cognitive Self-Consciousness	17.09
15	Emotional Bias	EGNG-Commission Rate HC	15.76
16	Emotional Bias	EGNG-Happy Effect False Alarms	15.52
17	Anhedonia	SHAPS	14.65
18	Attention	ANT-Conflict Effect	14.53
19	Aggression	BPAS-Total Score	13.75
20	Emotional Bias	EGNG-Fear Effect RT	19.28

## Discussion

The multi-dimensional assessment battery and ML analytics reveal significant insights into neurobehavioral factors associated with comorbid PTSD symptoms among patients with SUD. For participants with only self-report assessment data, the prediction algorithm failed to accurately predict a dimensional PCL-5 score, but did accurately classify individuals as having significant comorbid PTSD symptoms (or not), based on symptoms of anxiety and depression as well as perseverative/intrusive thought patterns, low tolerance of aversive body sensations, and reduced ability to focus on body sensations. In participants who also completed neurocognitive assessments, continuous PTSD symptom severity and significant comorbid PTSD symptoms were predicted with impressive accuracy. Many of the same predictors that contributed to regression algorithms were also included in the classification algorithms, but the neurocognitive subsample classification algorithm uniquely included increased response bias toward by fear-related social information evaluated with the Emotional Go-NoGo Task.

Our results support previous research linking comorbid SUD and PTSD to increased negative affect and cognitive biases. Elevated negative affect is a key feature of PTSD and is strongly associated with SUD, thus our observation that comorbid presentations are associated with increased anxiety and depression is logical. The current study also suggests that PTSD symptomatology in SUD patients is associated with a general deficit in regulating aversive internal states, whether cognitive (e.g., worrisome thoughts), affective (e.g., distress), or interoceptive (e.g., aversive body sensations) in nature. Indeed, this notion is consistent with observations that anxiety sensitivity and sensitivity to punishment are shared vulnerability factors [21, 23, 32]. Anxiety sensitivity reflects the propensity to react negatively or fearfully to anxiety-related sensations, thoughts, emotions, or environmental stimuli and has been linked to PTSD [57], SUD [58], comorbid SUD-PTSD [59], and increased responsivity to the fear-dampening effects of alcohol [60, 61]. Further, there is indeed robust evidence of increased attentional bias toward social threat in anxiety-related disorders [62–64] and PTSD [65]. Exaggerated attentional bias toward negative information in anxiety disorders is thought to stem from an imbalance between an exaggerated bottom-up processing of threat or insufficient top-down regulation of threat response by executive control neurocircuitry [66]; this bias is thought to alter subsequent steps of cognitive processing, such as promoting rumination [62]. Consequently, several clinical trials have attempted to reduce attentional bias to threat directly, wherein the patient is trained over multiple trials to disengage from threat stimuli such as by directing gaze to alternative stimuli [67]. Similar bias modification techniques have also targeted attentional capture by substances of abuse [68], including more “gamified” approaches to reduce monotony [69]. These findings collectively lead to the hypothesis that top-down regulation of attention may be bolstered by repeated bias training for one modality (e.g., threat) which may aid in reducing attentional capture by another trigger (e.g., drug-related stimuli).

This study supports existing findings and offers new insights into the relationship between SUD and PTSD. We anticipated that the addition of neurocognitive task scores to predict PTSD symptomatology would improve model performance and that scores from neurocognitive tasks would be utilized as splitting nodes in decision trees. This expectation stems from fact that the constructs measured by neurocognitive assessments, such as response inhibition, tend to be strongly associated with many mental illnesses [70, 71]. However, neurocognitive tasks may also be affected by transient states of fatigue or mental distraction, or conversely prone to uncharacteristically high vigilance summoned under artificial conditions. Self-report questionnaires, with the generally longer time span and real-life related contexts specified in question items, are thought to be more stable than performance on “one-shot” computerized cognitive tasks [72]. As such, it is possible that the presence of PTSD symptoms among SUD patients does not impede cognitive abilities in all contexts, but rather is associated with a subtle shift in cognition that can be functionally important and noticeable over time but are difficult to detect via computerized tests. Another noteworthy feature of our regression-based models was the indication that some constructs may be associated specifically with the extremes of post-traumatic stress symptom severity. For example, aggression-related constructs (from the Buss-Perry Aggression Scale) were only featured in regression algorithms as differentiating very high scores from moderately high scores on the PCL-5. These data may indicate that aggression is only a notable feature in SUD patients with severe PTSD symptoms. Accordingly, perhaps intervention strategies aimed at persons with SUD and mild-to-moderate comorbid PTSD should not be aimed at anger management or social functioning, whereas these may be important components of an intervention for severe comorbid PTSD.

In spite of these insights, the results should be considered in light of some limitations. First, because the study did not include a third group of participants with heightened PTSD symptoms alone (no SUD), we cannot conclude that the traits/risk factors identified truly differentiate comorbid SUD-PTSD from each disorder alone. The predictors highlighted in our results may be reflective of the increased overall disorder burden of comorbid conditions rather than SUD or PTSD alone [73]. Therefore, future studies will benefit from approaches that identify which psychopathological mechanisms differentiate comorbid SUD-PTSD from each disorder on its own. We also did not collect data on comorbid psychiatric diagnoses, which could have influenced study results and could be highly relevant for personalized treatment approaches. Additionally, while the inclusion of persons across multiple types of SUD can enable our predictive variables to serve as potential risk markers that cut across different types of SUDs, our results could presumably vary depending upon the primary substance of abuse. Indeed, individuals with more significant PTSD symptoms related to the hyperarousal symptom cluster were more likely to abuse alcohol, while those with more of avoidance and re-experiencing symptoms were more likely to abuse cocaine [74]. Future studies are therefore needed to compare data across multiple samples of patients who abuse different substances, including substance use disorders not examined in the current study (e.g., alcohol, methamphetamine) and polysubstance use disorders. Relatedly, heterogeneity among symptoms of PTSD may have impacted our findings, such that important subtypes of post-traumatic stress may be associated with unique phenotypic signatures. Multiple subtypes of PTSD have been proposed, including externalizing vs. internalizing subtypes as well as a dissociating subtype [75]. It is therefore possible that participants with comorbid PTSD symptoms were affected by distinct clusters of symptoms, which do not translate well into a linear models of disorder severity, and thus are not easily predicted by our ML approaches. While our predictive algorithms achieved relatively high accuracy, there is a clear need for future studies to deliver greater predictive accuracy by either increasing sample size or including additional biological predictors, particularly neurophysiological variables such as peripheral biomarkers, neuroimaging metrics, and genetic information.

Nevertheless, the performance of our models support a combination of self-report traits and neurocognitive abilities to be involved in the underlying etiology of comorbid SUD and PTSD. Our results from a machine-learning-based approach largely align with previous work, yet also provide a efficient integration of works investigating the role of a single risk factor or etiological feature. Future studies are planned to replicate these results and determine whether targeted treatments aimed at the identified psychopathological processes are associated with improved care for this high-need population.

### Supplementary information

Supplementary Material
