# Supplementary material for: Behavioral and neurocognitive factors distinguishing post-traumatic stress comorbidity in substance use disorders
Source: Transl Psychiatry. 2023 Sep 14;13:296. doi: 10.1038/s41398-023-02591-3 (PMC10502088; doi:10.1038/s41398-023-02591-3)
Supplement: Supplementary file 1 — Supplementary Material [file 41398_2023_2591_MOESM1_ESM.docx]

**SUPPLEMENTARY MATERIAL**

**Detailed Measure Descriptions**

*Cognition Measures:*

Backwards Digit Span (BDS). The BDS [1] is a cognitive task that measures working memory. Working memory deficits have been documented in a majority of persons with substance use disorders (SUDS) [2-5]. Participants are presented with a series of alphabetical digits and asked to repeat the sequence in reverse order. The length of digit sequences begins with two digits and grows by one digit upon successful responses, and adaptive testing allows for the difficulty of the task to be modulated based on responses across 14 trials. The typical outcome measure of the BDS is the number of digits at which the participant can recall correctly (in reverse order) before making two consecutive errors: two-error maximum length (bTE_ML). The number of trials completed as part of adaptive testing before two-error maximum length is reached is also often reported as a measure of consistency: two-error total trials (bTE_TT). Two-error total trials (bML) refers to the maximum number of digits that the participant could recall correctly over 14 trials, which reflects maximum working memory capacity. Mean backward digit span (bMS) refers to the number of digits that the participant would be expected to recall correctly 50% of the time, based on number of correct responses and number of errors during different digit sequence lengths.

Attention Network Task (ANT). The ANT [6] is a cognitive task that measures three attentional processes, or networks. In 120 trials, participants are asked to rapidly indicate whether a horizontal arrow that is briefly displayed on the screen (1000ms) is pointing right or left. The target arrow is presented on the upper or lower half of the screen alongside a series of flanker arrows that are oriented congruently (i.e., pointed in the same direction as the target) or incongruently (i.e., pointed in the opposite direction as the target). Target presentations are preceded by one of three cues: (1) no cue, (2) a center cue signaling that a target arrow will soon appear above or below the cue, (3) a spatial cue on the upper or lower half of the screen indicating that a target arrow will soon appear in that location. The Alerting Effect reflects the degree to which a person responds more quickly to targets when they are preceded by a cue that signals an impending target. The Orienting Effect outcome reflects the degree to which a person responds more quickly to a target if its location was previously signaled with a cue at that location. The Conflict Effect measures the degree to which reaction time increases when targets are presented alongside incongruent versus congruent flankers. Performance deficits on the ANT have been documented in persons with SUDs [7-9].

Stop-Signal Task (SST). The SST [10, 11] is an adaptive neurocognitive task designed to measure persons’ ability to inhibit actions once they are initiated. Poor response inhibition has been consistently associated with heightened risk for drug abuse behavior and substance use disorder [12]. Participants fixate on a center cross and are intermittently presented with arrows pointing horizontally left or right, at which time they are asked to press corresponding keys as quickly as possible. An auditory “stop-signal” is presented on some trials shortly after the “go” signal, during which time the participants must stop themselves from responding. The mean delay of stop signals across trials (SSD) and mean time required for a participant to stop (SSRT) are the primary outcome measures, and secondary outcomes include response times for commission errors (SR_RT), mean reaction times for correct responses (SR_NS), percent correct (NS_HIT), and number of omission errors (NS_MISS).

*Reward Measures:*

Short Urgency-Premeditation-Perseverance-Sensation Seeking-Positive Urgency (SUPPS) Impulsive Behavior Scale. The SUPPS [13] is a 20-item self-report questionnaire designed to measure various facets of impulsivity. Each facet of impulsivity measured by the scale is reflected in a sub-scale score. Negative urgency reflects the tendency to act impulsively under conditions of negative affect. High scores on the Premeditation subscale reflect tendency to act without consideration of the consequences of one’s behavior. High scores on the Perseverance subscale reflect a tendency to give up easily on tasks when faced by boredom, fatigue, and/or frustration. Sensation seeking reflects individuals’ interest in and tendency to pursue activities that are exciting and novel. Positive urgency reflects a tendency to act impulsively under conditions of high positive affect. Scores on the S-UPPS have been consistently associated with substance use [14, 15] and substance use disorders [16, 17].

5-Trial Delay Discounting Task (5DD). The 5DD [18] is a brief and adaptive test designed to measure delay discounting, or the propensity to discount larger awards that can be accessed later in favor of smaller, immediate rewards. Heightened levels of delay discounting have been consistently linked to SUDs and similar impulsive behavior disorders [19]. Modeled after a lengthy questionnaire [20], participants are asked to choose whether they would have a relatively small amount of money now or a relatively larger amount of money on a later date, with the sizes of the monetary amounts and time delays varying for each question.

*Interoception Measures:*

Multidimensional Assessment of Interoceptive Awareness (MAIA). The MAIA [21] is a 32-item self-report questionnaire designed to measure various facets of interoception. The Noticing subscale reflects awareness of body sensations. Scores on the Not Distracting substance reflect a lack of distress or worry in response to aversive body sensations. Scores on the Not Worrying subscale reflect one’s appraisal of aversive bodily sensations. Scores on the Attention Regulation subscale reflect ability to focus on body sensations. Scores on the Emotional Awareness Subscale reflect increased recognition of the connection between body sensations and emotional states. High scores on the Self-Regulation subscale reflect increased ability to modulate emotion in response to body sensations. The Body Listening subscale reflects tendency to focus on body sensations to determine how one is feeling. Finally, the Trusting subscale reflects overall feeling of comfort and safety in one’s body. Abnormal interoception has been reported in various psychopathologies, including substance use disorders [22-26], but there is very little research with the MAIA on substance use disorders.

*Negative Emotionality Measures:*

Emotional Go-NoGo Task (EGNG). The EGNG [27] is a cognitive task that measures the degree to which attentional bias toward emotionally-valanced face images as either targets or non-targets either disrupts or facilitates behavioral responding. During each block of the EGNG, participants view a series of photographs of actor faces, each displaying either fear, happiness, or calmness (i.e., no emotion). Within specific blocks, participants are asked to press a key whenever a face appears that expresses a certain emotion but to withhold responses to faces showing another (non-target) emotion. Attentional bias is reflected in the degree to which faces displaying certain emotions facilitate deviations in average response times and response accuracy when used as target stimuli (e.g., response time decreases when “happy” faces are targets) or distractor stimuli (e.g., more incorrect key presses to “fear” faces as distractors). We utilized the original version of the EGNG [27] with only fear-vs-calm and happy-vs-calm blocks with non-target no-go-stimuli presented in 25% of trials. There were four task blocks of 48 trials each. Rates of correct responses (hits to targets), commission errors (hits to non-targets) and median reaction time to targets (hits) were computed within each block and overall. In addition, omnibus signal detection statistics were computed: A is a non-parametric statistic of sensitivity (overall performance accuracy), whereas b (log-transformed for normality) reflects response bias (conservative versus liberal). Finally, to determine whether certain emotional faces capture attention more strongly, a “happy effect” and “fear effect” were calculated by comparing reaction times when that emotion was target face type versus when that emotion type was the non-target face type. Higher values mean faster responding to that face/emotion type as a target.

Distress Tolerance Scale (DTS). The DTS [28] is a 15-item self-report measure of emotional distress tolerance. The measure provides a global index of distress tolerance as well as four subscales: tolerance, absorption, appraisal, and regulation. Scores on the tolerance subscale reflect one’s ability to tolerate aversive emotions. Scores on the absorption subscale reflect the tendency for negative affective states to dominate attention and disrupt functioning. Scores on the appraisal subscale reflect one’s acceptance of negative affective states. Scores on the regulation subscale reflect one’s ability to modulate emotions as well as their tendency to avoid or immediately attempt to escape from aversive emotions. Low levels of distress tolerance have consistently been linked to substance use disorders [29-31].

Patient-Reported Outcomes Measurement Information System – Depression scale (PROMIS-D). The PROMIS-D [32] is a 4-item self-report questionnaire that assesses depression severity. This brief questionnaire has comparable psychometric properties with longer depression severity measures.

Patient-Reported Outcomes Measurement Information System – Anxiety Scale (PROMIS-A). The PROMIS-A [32] is a 4-item self-report questionnaire that assesses anxiety severity. Similar to the PROMIS-D, this brief measure has good psychometric properties.

Buss-Perry Aggression Scale (BPAS). The BPAS [33] is a 29-item self-report questionnaire that measures various facets of aggression. A total score is provided that reflects overall aggressive behavioral tendencies and personality characteristics. The four subscales reflect physical aggression, verbal aggression, anger, and hostility (i.e., paranoia, passive aggression). High levels of substance use and substance use disorders have consistently been linked to increases in violent behavior and aggression [34, 35].

Snaith-Hamilton Pleasure Scale (SHAPS). The SHAPS [36] is a 14-item self-report questionnaire that measures anhedonia, or the inability to experience pleasure. A significant body of evidence has shown that levels of anhedonia are often elevated amongst persons with SUDS [37].

Metacognitions Questionnaire-30 (MCQ). The MCQ [38] is a 30-item self-report questionnaire designed to measure several higher-order beliefs that are believed to be related to psychopathology. These higher-order beliefs include positive beliefs about worry (e.g., “My worry is useful”), a need to control thoughts, negative beliefs about thoughts concerning uncontrollability and danger, cognitive confidence (i.e., trust in one’s memory), and cognitive self-confidence (i.e., degree of self-monitoring of one’s own thoughts). Each belief is represented by a subscale, and the total score reflects overall level of perseverative thinking, biased attention, and ineffective self-regulation strategies. High scores on the MCQ-30 total score and its subscales have been linked to substance use disorders [39, 40].

Pittsburgh Sleep Quality Index (PSQI). The PSQI [41] is a 19-item questionnaire that measures the overall quality of nighttime sleep in terms of sleep latency, sleep duration, sleep disturbance, restfulness, and daytime sleepiness. For the purposes of this study, the global index of overall sleep quality was used. Sleep disturbances have been documented among persons with SUDS [42, 43].

**Supplementary Table 1. Confusion Matrix for TreeNet Training Data – Full Sample**

|  | Predicted Group | |  |
| --- | --- | --- | --- |
| Actual Group | No/Low Trauma | Moderate/Severe Trauma | Total |
| No/Low Trauma | 84 | 9 | 93 |
| Moderate/Severe Trauma | 1 | 38 | 39 |
| Total | 85 | 47 | 132 |

**Supplementary Table 2. Confusion Matrix for TreeNet Testing Data – Full Sample**

|  | Predicted Group | |  |
| --- | --- | --- | --- |
| Actual Group | No/Low Trauma | Moderate/Severe Trauma | Total |
| No/Low Trauma | 12 | 6 | 18 |
| Moderate/Severe Trauma | 2 | 8 | 10 |
| Total | 14 | 14 | 28 |

**Supplementary Table 3. Confusion Matrix for CART Training Data – Full Sample**

|  | Predicted Group | |  |
| --- | --- | --- | --- |
| Actual Group | No/Low Trauma | Moderate/Severe Trauma | Total |
| No/Low Trauma | 82 | 11 | 93 |
| Moderate/Severe Trauma | 1 | 38 | 39 |
| Total | 83 | 49 | 132 |

**Supplementary Table 4. Confusion Matrix for CART Testing Data – Full Sample**

|  | Predicted Group | |  |
| --- | --- | --- | --- |
| Actual Group | No/Low Trauma | Moderate/Severe Trauma | Total |
| No/Low Trauma | 13 | 5 | 18 |
| Moderate/Severe Trauma | 2 | 8 | 10 |
| Total | 15 | 13 | 28 |

**Supplementary Table 5. Confusion Matrix for TreeNet Training Data – Neurocognitive Sub-Sample**

|  | Predicted Group | |  |
| --- | --- | --- | --- |
| Actual Group | No/Low Trauma | Moderate/Severe Trauma | Total |
| No/Low Trauma | 46 | 12 | 58 |
| Moderate/Severe Trauma | 0 | 29 | 29 |
| Total | 46 | 41 | 87 |

**Supplementary Table 6. Confusion Matrix for TreeNet Testing Data – Neurocognitive Sub-Sample**

|  | Predicted Group | |  |
| --- | --- | --- | --- |
| Actual Group | No/Low Trauma | Moderate/Severe Trauma | Total |
| No/Low Trauma | 9 | 3 | 12 |
| Moderate/Severe Trauma | 1 | 2 | 3 |
| Total | 10 | 5 | 15 |

**Supplementary Table 7. Confusion Matrix for CART Training Data – Neurocognitive Sub-Sample**

|  | Predicted Group | |  |
| --- | --- | --- | --- |
| Actual Group | No/Low Trauma | Moderate/Severe Trauma | Total |
| No/Low Trauma | 50 | 8 | 58 |
| Moderate/Severe Trauma | 1 | 28 | 29 |
| Total | 51 | 36 | 87 |

**Supplementary Table 8. Confusion Matrix for CART Testing Data – Neurocognitive Sub-Sample**

|  | Predicted Group | |  |
| --- | --- | --- | --- |
| Actual Group | No/Low Trauma | Moderate/Severe Trauma | Total |
| No/Low Trauma | 11 | 1 | 12 |
| Moderate/Severe Trauma | 1 | 2 | 3 |
| Total | 12 | 3 | 15 |

**Supplementary Figure 1. Effect Sizes of Predictors’ Association with Binary PCL-5 Outcome – Full Sample.**

**Supplementary Figure 2. Effect Sizes of Predictors’ Association with Binary PCL-5 Outcome – Neurocognitive Sub-Sample.**

**Supplementary Figure 3. Receiver Operating Characteristic Curves for TreeNet Analysis – Full Sample.** Solid red line represents training data. Dashed blue line represents testing data.


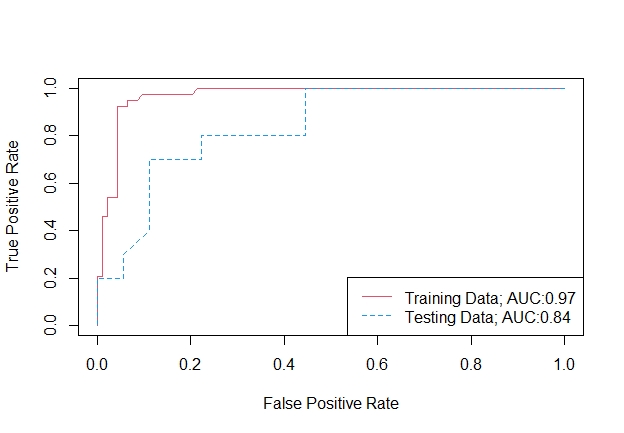


**Supplementary Figure 4. Receiver Operating Characteristic Curves for CART Analysis – Full Sample.** Solid red line represents training data. Dashed blue line represents testing data.


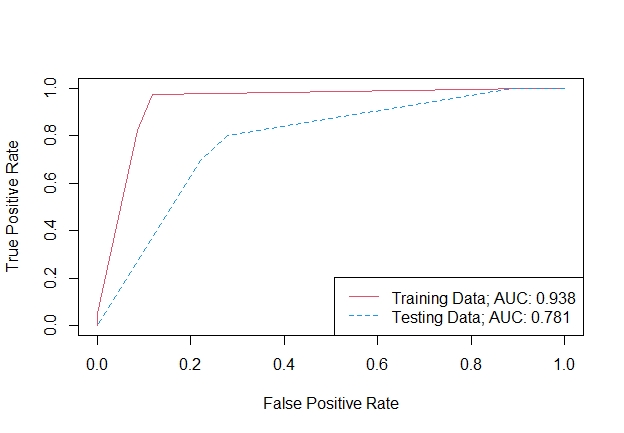


**Supplementary Figure 5. Receiver Operating Characteristic Curves for TreeNet Analysis – Neurocognitive Sub-Sample.** Solid red line represents training data. Dashed blue line represents testing data.

**
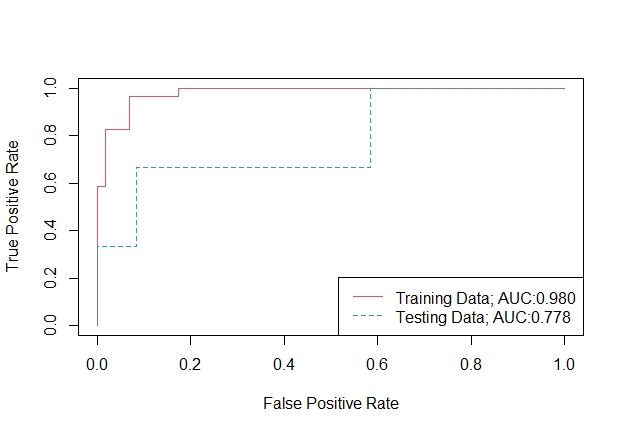
**

**Supplementary Figure 6. Receiver Operating Characteristic Curves for CART Analysis – Neurocognitive Sub-Sample.** Solid red line represents training data. Dashed blue line represents testing data.


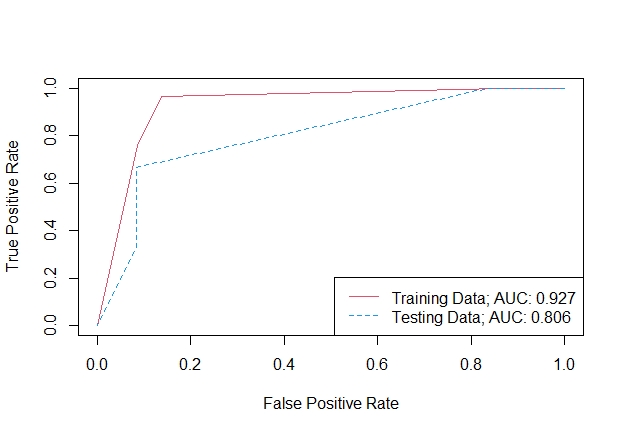


References:

1. Conway AR, Kane MJ, Bunting MF, Hambrick DZ, Wilhelm O, & Engle RW, Working memory span tasks: A methodological review and user's guide. Psychon Bull Rev 2005; 12(5): 769-786.

2. Aharonovich E, Hasin DS, Brooks AC, Liu X, Bisaga A, & Nunes EV, Cognitive deficits predict low treatment retention in cocaine dependent patients. Drug Alcohol Depend 2006; 81(3): 313-322.

3. Bates ME, Pawlak AP, Tonigan JS, Buckman JF, Cognitive impairment influences drinking outcome by altering therapeutic mechanisms of change. Psychol Addict Behav 2006; 20(3): 241-253.

4. Goldman MS, Experience-dependent neuropsychological recovery and the treatment of chronic alcoholism. Neuropsychol Rev 1990; 1(1): 75-101.

5. Gonzalez R, Rippeth JD, Carey CL, Heaton RK, Moore DJ, Schweinsburg BC, *et al*. Neurocognitive performance of methamphetamine users discordant for history of marijuana exposure. Drug Alcohol Depend 2004; 76(2): 181-190.

6. Fan J, McCandliss BD, Sommer T, Raz A, & Posner MI, Testing the efficiency and independence of attentional networks. J Cogn Neurosci 2002; 14(3): 340-347.

7. Lannoy S, Heeren A, Moyaerts N, Bruneau N, Evrard S, Billieux J, & Maurage P, Differential impairments across attentional networks in binge drinking. Psychopharmacology 2017; 234(7): 1059-1068.

8. Salo R, Gabay S, Fassbender C, & Henik A, Distributed attentional deficits in chronic methamphetamine abusers: evidence from the Attentional Network Task (ANT). Brain Cogn 2011; 77(3): 446-452.

9. Vilar-Lopez R, Takagi M, Lumban DI, Cotton SM, Bora E, Verdejo-Garcia A, & Yuecel M, The effects of inhalant misuse on attentional networks*.* Dev Neuropsychol 2013; 38(2): 126-136.

10. Verbruggen F, Aron AR, Band GP, Beste C, Bissett P, Brockett AT, *et al*. A consensus guide to capturing the ability to inhibit actions and impulsive behaviors in the stop-signal task. Elife 2019; 8: e46323.

11. Verbruggen F, Logan GD, & Stevens MA, STOP-IT: Windows executable software for the stop-signal paradigm. Behav Res Methods 2008; 40(2): 479-483.

12. Perry JL, & Carroll ME, The role of impulsive behavior in drug abuse. Psychopharmacology 2008; 200(1): 1-26.

13. Cyders MA, Littlefield AK, Coffey S, & Karyadi KA, Examination of a short English version of the UPPS-P Impulsive Behavior Scale. Addict Behav 2014; 39(9): 1372-1376.

14. Sanchez-Roige S, Fontanillas P, Elson SL, Gray JC, de Wit H, MacKillop J, *et al*. Genome-Wide Association Studies of Impulsive Personality Traits (BIS-11 and UPPS-P) and Drug Experimentation in up to 22,861 Adult Research Participants Identify Loci in the CACNA1I and CADM2 genes. J Neurosci 2019; 39(13): 2562-2572.

15. Robinson E, Jones A, Christiansen P, & Field M, Drinking like everyone else: trait self-control moderates the association between peer and personal heavy episodic drinking. Subst Use Misuse 2015, 50(5): 590-597.

16. Gunn RL, Jackson KM, Borsari B, & Metrik J, Negative urgency partially accounts for the relationship between major depressive disorder and marijuana problems. Borderline Personal Disord Emot Dysregul 2018; 5: 1-8.

17. Mirhashem R, Allen HC, Adams ZW, van Stolk-Cooke K, Legrand A, & Price M, The intervening role of urgency on the association between childhood maltreatment, PTSD, and substance-related problems. Addict Behav 2017; 69: 98-103.

18. Koffarnus MN, & Bickel WK, A 5-trial adjusting delay discounting task: accurate discount rates in less than one minute. Exp Clin Psychopharmacol 2014; 22(3): 222-228.

19. Reynolds B, A review of delay-discounting research with humans: relations to drug use and gambling. Behav Pharmacol 2006; 17(8): 651-67.

20. Madden GJ, Petry NM, Badger GJ, & Bickel WK, Impulsive and self-control choices in opioid-dependent patients and non-drug-using control participants: drug and monetary rewards. Exp Clin Psychopharmacol 1997; 5(3): 256-262.

21. Mehling WE, Acree M, Stewart A, Silas J, & Jones A, The Multidimensional Assessment of Interoceptive Awareness (MAIA). PLoS One 2012; 7(11): e48230.

22. Avery JA, Burrows K, Kerr KL, Bodurka J, Khalsa SS, Paulus MP, & Simmons WK, How the Brain Wants What the Body Needs: The Neural Basis of Positive Alliesthesia. Neuropsychopharmacology 2017; 42(4): 822-830.

23. Goldstein RZ, Bechara A, Garavan H, Childress AR, Paulus MP, & Volkow ND, The neurocircuitry of impaired insight in drug addiction. Trends Cogn Sci 2009; 13(9): 372-380.

24. Khalsa SS, Adolphs R, Cameron OG, Critchley HD, Davenport PW, Feinstein JS, *et al*. Interoception and Mental Health: A Roadmap. Biol Psychiatry Cogn Neurosci Neuroimaging 2018; 3(6): 501-513.

25. Naqvi NH, & Bechara A, The hidden island of addiction: the insula. Trends Neurosci 2009; 32(1): 56-67.

26. Paulus MP, Stewart JL, & Haase L, Treatment approaches for interoceptive dysfunctions in drug addiction. Front Psychiatry 2013; 4: 137.

27. Tottenham N, Hare TA, & Casey BJ, Behavioral assessment of emotion discrimination, emotion regulation, and cognitive control in childhood, adolescence, and adulthood. Front Psychol 2011; 2: 39.

28. Simons JS, & Gaher RM, The Distress Tolerance Scale: Development and validation of a self-report measure. Motivation and Emotion 2005; 29(2): 83-102.

29. Allan NP, Macatee RJ, Norr AM, Raines AM, & Schmidt NB, Relations between common and specific factors of anxiety sensitivity and distress tolerance and fear, distress, and alcohol and substance use disorders. J Anxiety Disord 2015; 33: 81-89.

30. Buckner JD, Keough ME, & Schmidt NB, Problematic alcohol and cannabis use among young adults: the roles of depression and discomfort and distress tolerance. Addict Behav 2007; 32(9): 1957-1963.

31. Shorey RC, Gawrysiak MJ, Elmquist J, Brem M, Anderson S, & Stuart GL, Experiential avoidance, distress tolerance, and substance use cravings among adults in residential treatment for substance use disorders. J Addict Dis 2017; 36(3): 151-157.

32. Cella D, Riley W, Stone A, Rothrock N, Reeve B, Yount S, *et al*. The Patient-Reported Outcomes Measurement Information System (PROMIS) developed and tested its first wave of adult self-reported health outcome item banks: 2005-2008. J Clin Epidemiol 2010; 63(11): 1179-1194.

33. Buss AH, & Perry M, The aggression questionnaire. J Pers Soc Psychol 1992; 63(3): 452-459.

34. Barrett EL, Teesson M, & Mills KL, Associations between substance use, post-traumatic stress disorder and the perpetration of violence: A longitudinal investigation. Addict Behav 2014; 39(6): 1075-1080.

35. Krueger RF, Markon KE, Patrick CJ, Benning SD, & Kramer MD, Linking antisocial behavior, substance use, and personality: an integrative quantitative model of the adult externalizing spectrum. J Abnorm Psychol 2007; 116(4): 645-666.

36. Snaith RP, Hamilton M, Morley S, Humayan A, Hargreaves D, & Trigwell P, A scale for the assessment of hedonic tone the Snaith-Hamilton Pleasure Scale. Br J Psychiatry 1995; 167(1): 99-103.

37. Garfield JB, Lubman DI, & Yucel M, Anhedonia in substance use disorders: a systematic review of its nature, course and clinical correlates*.* Aust N Z J Psychiatry 2014; 48(1): 36-51.

38. Wells A, & Cartwright-Hatton S, A short form of the metacognitions questionnaire: properties of the MCQ-30. Behav Res Ther 2004; 42(4): 385-396.

39. Hamonniere T, Laqueille X, Vorspan F, Dereux A, Illel K, & Vareson I, Toward a better understanding of the influence of repetitive negative thinking in alcohol use disorder: An examination of moderation effect of metacognitive beliefs and gender. Addict Behav 2020; 111: 106561.

40. Hamonniere T, & Varescon I, Metacognitive beliefs in addictive behaviours: A systematic review. Addict Behav 2018; 85: 51-63.

41. Buysse DJ, Reynolds III CF, Monk TH, Berman SR, & Kupfer DJ, The Pittsburgh Sleep Quality Index: a new instrument for psychiatric practice and research. Psychiatry Res 1989; 28(2): 193-213.

42. Chakravorty S, Chaudhary NS, Brower KJ, Alcohol Dependence and Its Relationship With Insomnia and Other Sleep Disorders. Alcohol Clin Exp Res 2016; 40(11): 2271-2282.

43. Lind MJ, Baylor A, Overstreet CM, Hawn SE, Rybarczyk BD, Kendler KS, *et al*. Relationships between potentially traumatic events, sleep disturbances, and symptoms of PTSD and alcohol use disorder in a young adult sample. Sleep Med 2017; 34: 141-147.
